# Circle of Willis Anatomical Variations on CT Angiography: Prevalence and Clinical Associations in a Turkish Cohort

**DOI:** 10.3390/medicina62101994

**Published:** 2026-10-15

**Authors:** Bekir Sıtkı Ulusoy, Duygu Aslan, Ahmet Burak Kara, Mehmet Cudi Tuncer

**Affiliations:** 1 Department of Radiology, Ayfonkarahisar State Hospital, Afyonkarahisar 03030, Turkey; 2 Department of Anatomy, Medical School, Dicle University, Diyarbakır 21280, Turkey; drduyguaslan@outlook.com (D.A.); drcudi@hotmail.com (M.C.T.); 3 Department of Radiology, Gaziantep City Hospital, Gaziantep 27470, Turkey; ahmetburakkara@gmail.com

**Keywords:** Circle of Willis, anatomical variants, computed tomography angiography, cerebral circulation, posterior communicating artery, cerebrovascular anatomy

## Abstract

*Background and Objectives:* Anatomical variations in the Circle of Willis (CoW) may influence cerebral collateral pathways and the interpretation of vascular imaging. This study characterized their prevalence and clinical associations in a Turkish computed tomography angiography (CTA) cohort using complementary patient-level and component-level classifications. *Materials and Methods:* This retrospective, single-center study included 248 patients who underwent cranial CTA during 2024. Two radiologists independently assessed CoW anatomy, with disagreements resolved by consensus. Mutually exclusive patient-level configurations and non-exclusive arterial components were recorded. Associations with demographic and clinical characteristics were evaluated using logistic regression. *Results:* A normal configuration was identified in 142 patients (57.3%), and 106 (42.7%) had at least one variant. Isolated posterior communicating artery (PComA) hypoplasia/aplasia was the most frequent configuration (14.5%); PComA involvement reached 19.0% when mixed configurations were included. Isolated fetal-type posterior cerebral artery, P1 hypoplasia, and A1 hypoplasia occurred in 7.3%, 6.5%, and 4.8%, respectively. Older age (adjusted odds ratio [aOR] per 10 years, 1.57; 95% confidence interval [CI], 1.23–2.01) and migraine (aOR, 5.67; 95% CI, 2.06–15.56) were associated with variant anatomy. Previous stroke/transient ischemic attack and carotid stenosis showed inverse associations. *Conclusions:* CoW variants were common in this Turkish clinical cohort, particularly in the posterior circulation. Reporting both overall configurations and individual arterial components provides complementary information relevant to anatomical assessment, vascular imaging interpretation, and procedural planning.

## 1. Introduction

The CoW is a major arterial anastomotic network at the base of the brain that connects the anterior and posterior cerebral circulations and provides potential pathways for collateral blood flow. Although the classical configuration serves as an anatomical reference, considerable interindividual variation exists in the morphology, caliber, and continuity of its arterial segments [1,2]. Recognition of these variations is relevant to cerebrovascular imaging interpretation and neurovascular procedural planning.

Several classification systems have been developed to describe CoW anatomy. Earlier approaches, including those proposed by Riggs and Rupp, Lazorthes et al., and Krabbe-Hartkamp et al., differ in their anatomical scope and definitions of hypoplastic or absent arterial segments [2,3,4]. More recently, a systematic review catalogued 82 variants and proposed a comprehensive classification and coding framework [5]. Complementary approaches classify the anterior and posterior components according to their completeness [2,6]. These differences highlight the need for consistent terminology and explicit reporting of individual arterial components and mixed configurations.

The clinical relevance of CoW variations has been investigated in migraine and cerebrovascular disease. Incomplete configurations have been reported more frequently in individuals with migraine with aura [7], while other studies have examined associations between CoW anatomy and ischemic stroke characteristics and outcomes [8,9,10]. However, these relationships are complex, and associations identified in observational studies should not be interpreted as evidence of direct causality.

Several imaging modalities can characterize CoW anatomy. Magnetic resonance angiography (MRA) enables non-invasive assessment without ionizing radiation, CTA provides rapid visualization with high spatial resolution, and invasive digital subtraction angiography (DSA) is generally reserved for selected diagnostic and interventional indications [11,12,13]. Functional approaches, including dynamic arterial spin labeling MRI and transcranial Doppler (TCD), can provide complementary information on blood flow patterns and intracranial hemodynamics. Anatomical completeness does not necessarily correspond to functional collateral capacity.

Reported prevalence varies considerably across populations and methods. A Chinese study reported a complete anterior CoW in approximately 79% of individuals [14], whereas cadaveric studies have reported widely varying rates of whole-circle completeness [15,16]. PComA hypoplasia or aplasia is particularly common, with reported frequencies of approximately 34–68% in some populations [1,17], and anterior circulation variants also show population-specific differences [18]. A systematic review and meta-analysis estimated approximately 1.38-fold higher odds of ischemic stroke with incomplete CoW anatomy, although the pooled association was not statistically significant [9]. Differences in definitions and study design limit direct comparisons.

Regional studies provide important context. A Turkish CTA study of 100 adults reported a complete polygonal configuration in 28%, whereas a Turkish cadaveric study of 100 specimens identified a configuration without hypoplasia in 8% [12,16]. The Norwegian Tromsø Study reported a complete CoW in 11.9% of 1864 community participants aged 40–87 years using 3T time-of-flight MRA and a 1-mm diameter threshold [19]. The approximately 79% prevalence in the Chinese CTA series refers only to anterior-component completeness and is not directly comparable with whole-circle normality [14]. Differences in imaging modality, vessel-diameter thresholds, classification criteria, age distribution, and clinical selection must therefore be considered before attributing between-study variation to population background. The present study describes a Turkish clinical referral cohort and does not establish a nationally representative or ethnicity-specific anatomical pattern.

The contribution of this study is the combined reporting of mutually exclusive patient-level configurations and non-exclusive arterial component frequencies, together with adjusted demographic and clinical associations. This approach makes the handling of mixed configurations explicit and allows overall variant prevalence to be interpreted alongside the arterial components involved. We aimed to characterize CoW anatomy on cranial CTA in 248 patients from a Turkish clinical setting and to explore associations with age, sex, vascular risk factors, migraine, previous stroke/TIA, and carotid stenosis.

## 2. Materials and Methods

### 2.1. Study Design and Ethical Considerations

This retrospective, single-center observational study was conducted in the Department of Radiology of a tertiary hospital. Cranial CTA examinations performed for clinical indications between January 2024 and December 2024 were retrospectively evaluated. The study protocol was approved by the Afyonkarahisar Provincial Health Directorate Non-Interventional Clinical Research Ethics Committee (approval no. 2026/22; 11 June 2026), and retrospective screening and analysis of existing clinical and imaging records for research purposes were conducted only after ethics committee approval. At the time of CTA examination, patients provided written informed consent for the clinically indicated CT/CTA procedure, including contrast administration where applicable; this consent was related to the routine diagnostic procedure and did not constitute study-specific research consent. The study was conducted in accordance with the principles of the Declaration of Helsinki.

### 2.2. Patient Selection

**Figure 1:**
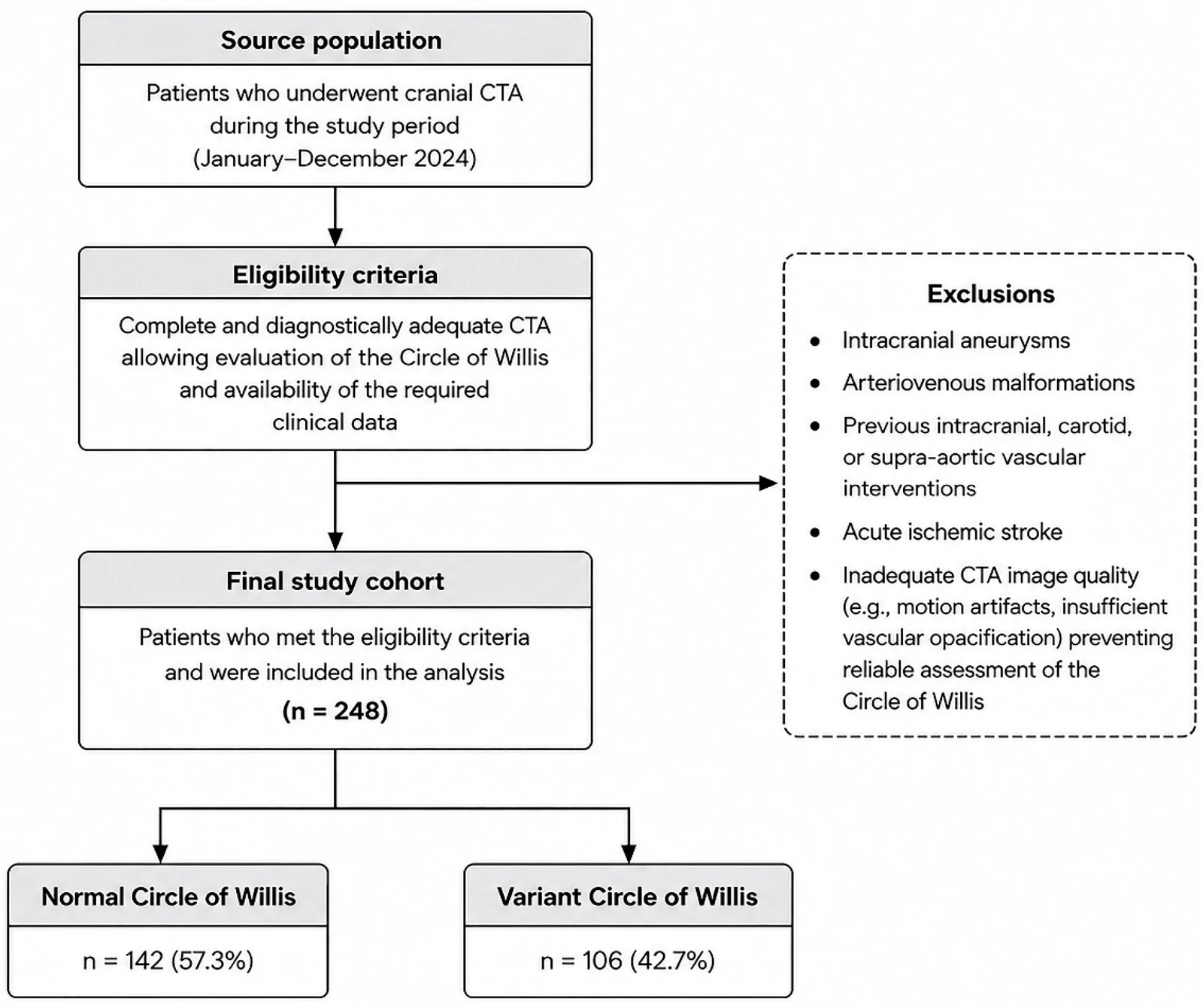
Flowchart of patient selection and classification according to CoW configuration. CTA, computed tomography angiography; CoW, Circle of Willis.

Patients who underwent cranial CTA during the study period were considered eligible for inclusion if the examination was complete and of sufficient diagnostic quality to allow reliable assessment of the CoW. Additional inclusion criteria were adequate visualization of the major arterial components of the CoW and availability of the demographic and clinical data required for the planned analyses. Patients with intracranial aneurysms, arteriovenous malformations, previous intracranial, carotid, or supra-aortic vascular interventions, acute ischemic stroke at the time of imaging, or technically inadequate CTA examinations due to motion artifacts, insufficient vascular opacification, or other factors precluding reliable evaluation of the CoW were excluded. After application of these eligibility criteria, 248 patients were included in the final study cohort (Figure 1). The cohort included pediatric and adult patients, with ages ranging from 0 to 94 years.

### 2.3. Imaging Protocol

Cranial CTA examinations were performed using a 256-slice multidetector CT scanner (SOMATOM Definition Flash, Siemens Healthcare, Erlangen, Germany). Acquisition parameters were 120 kVp, 35 mAs, 0.6 mm collimation, and a helical pitch of 0.8. A total of 80–100 mL of nonionic iodinated contrast medium (iohexol, 350 mg I/mL) was administered intravenously through an antecubital vein using a power injector at a flow rate of 4–5 mL/s, followed by a 40 mL saline flush. Image acquisition was initiated using an automated bolus-tracking technique, with the region of interest positioned in the ascending aorta. The scan coverage extended from the foramen magnum to the vertex.

### 2.4. Image Reconstruction and Analysis

All CTA images were reconstructed at a slice thickness of 0.6 mm and evaluated on a dedicated workstation using multiplanar reconstruction (MPR) and maximum intensity projection (MIP) techniques. Two board-certified radiologists independently reviewed the CTA examinations and assessed the arterial anatomy of the CoW in the axial, sagittal, and coronal planes. In cases of disagreement regarding the presence or classification of an anatomical variant, the images were jointly reviewed by both radiologists, and the final classification was determined by consensus.

### 2.5. Classification of CoW Variants

Based on the CTA findings, CoW configurations were classified into 10 predefined anatomical categories: (1) normal configuration; (2) hypoplasia or aplasia of the PComA; (3) fetal-type PCA; (4) hypoplasia or aplasia of the AComA; (5) azygos anterior cerebral artery (ACA); (6) ACA trifurcation; (7) A1 segment hypoplasia; (8) P1 segment hypoplasia; (9) vascular duplication, including duplication of the AComA or middle cerebral artery (MCA); and (10) other rare or combined configurations that did not fit the preceding categories.

Vessel status was determined according to visual continuity and luminal diameter on CTA. A vessel with a luminal diameter < 1 mm was classified as hypoplastic, whereas a vessel that was not visualized on the reconstructed CTA images was classified as aplastic. In this imaging-based classification, aplasia denotes non-visualization on CTA and does not establish congenital absence independently of spatial resolution, contrast opacification, or acquired vascular narrowing.

### 2.6. Data Coding and Outcome Definition

For statistical analysis, CTA findings were standardized into a mutually exclusive patient-level classification consisting of normal configuration, isolated PComA hypoplasia/aplasia, isolated fetal-type PCA, isolated A1 hypoplasia, isolated P1 hypoplasia, and other/mixed variants. Each patient was assigned to only one of these categories. Rare isolated variants and configurations containing more than one anatomical variant were grouped under the other/mixed category.

In addition, a complementary non-exclusive component-level classification was used to characterize the overall prevalence of individual anatomical variants. Under this approach, patients with mixed configurations contributed to each arterial component involved. The primary binary outcome for subsequent analyses was defined as the presence of any CoW variant (1) versus a normal CoW configuration (0).

Clinical comorbidities, migraine, and previous stroke/TIA were analyzed as binary history variables. Carotid stenosis was defined as ≥50% luminal narrowing of either internal carotid artery, as documented in imaging reports available in the medical records before or at the time of the index CTA, and was coded as a binary variable. Carotid stenosis was not determined from the cranial CTA used for CoW assessment, whose scan coverage extended from the foramen magnum to the vertex. Previous stroke and TIA were combined; separate associations by stroke type, vascular territory, or mechanism were not evaluated. Pharmacotherapy was not included among the reported analytical variables. Consequently, the analyses do not provide estimates adjusted for medication class, dose, treatment duration, or timing relative to CTA.

### 2.7. Statistical Analysis

Categorical variables were summarized as frequencies and percentages. Age was summarized as the median and interquartile range (IQR), with the mean and standard deviation (SD) additionally reported for descriptive purposes. Comparisons between patients with normal and variant CoW configurations were performed using the Mann–Whitney U test for age and Pearson’s chi-square test for categorical variables. For exploratory comparisons across the six mutually exclusive CoW configuration groups, age was analyzed using the Kruskal–Wallis test, whereas categorical variables were evaluated using the Fisher–Freeman–Halton exact test with 100,000 Monte Carlo samples. These exploratory comparisons comprised nine global tests: one for age and eight for categorical variables. To account for multiplicity within this family, a conservative Bonferroni significance threshold of 0.05/9 (approximately 0.0056) was applied to the reported global *p*-values. The tabulated *p*-values themselves were retained, and no post hoc pairwise comparisons were performed. These configuration-specific analyses remained exploratory.

Univariate and multivariable binary logistic regression analyses were performed to evaluate associations between demographic and clinical variables and the presence of any CoW variant. Results were reported as odds ratios (ORs) with 95% confidence intervals (CIs). Age was entered as a continuous variable scaled per 10 years, implying a common linear association with the log odds across the observed age range. The multivariable model simultaneously included age, sex, previous stroke/TIA, migraine, carotid stenosis, diabetes mellitus, hypertension, smoking, and hyperlipidemia. Multicollinearity among covariates was assessed using variance inflation factors (VIFs).

Model discrimination was assessed using the area under the receiver operating characteristic curve (AUC), with a bootstrap-derived 95% CI for the apparent AUC. Internal assessment of discrimination was additionally performed using 10-fold cross-validation. Model calibration and overall performance were evaluated using the Hosmer–Lemeshow goodness-of-fit test and Brier score, respectively, and model explanatory performance was summarized using pseudo-R^2^ measures. All statistical tests were two-sided. The nine configuration-specific comparisons were evaluated against the Bonferroni threshold, reported *p*-values were interpreted at the nominal 0.05 level. Statistical analyses were performed using Python version 3.13 with SciPy version 1.17.0, statsmodels version 0.14.6, and scikit-learn version 1.8.0.

## 3. Results

### 3.1. Baseline Characteristics and Variant Distribution

A total of 248 patients were included in the primary analysis. The median age was 47.0 years (IQR, 36.0–54.0), and the mean age was 45.7 ± 15.9 years; the age range was 0–94 years, including pediatric and adult patients. Of the 248 patients, 146 (58.9%) were male. A history of stroke/TIA was recorded in 109 patients (44.0%), hypertension in 74 (29.8%), hyperlipidemia in 68 (27.4%), smoking in 55 (22.2%), carotid stenosis in 49 (19.8%), migraine in 33 (13.3%), and diabetes mellitus in 25 (10.1%) (Table 1).

**Figure 2:**
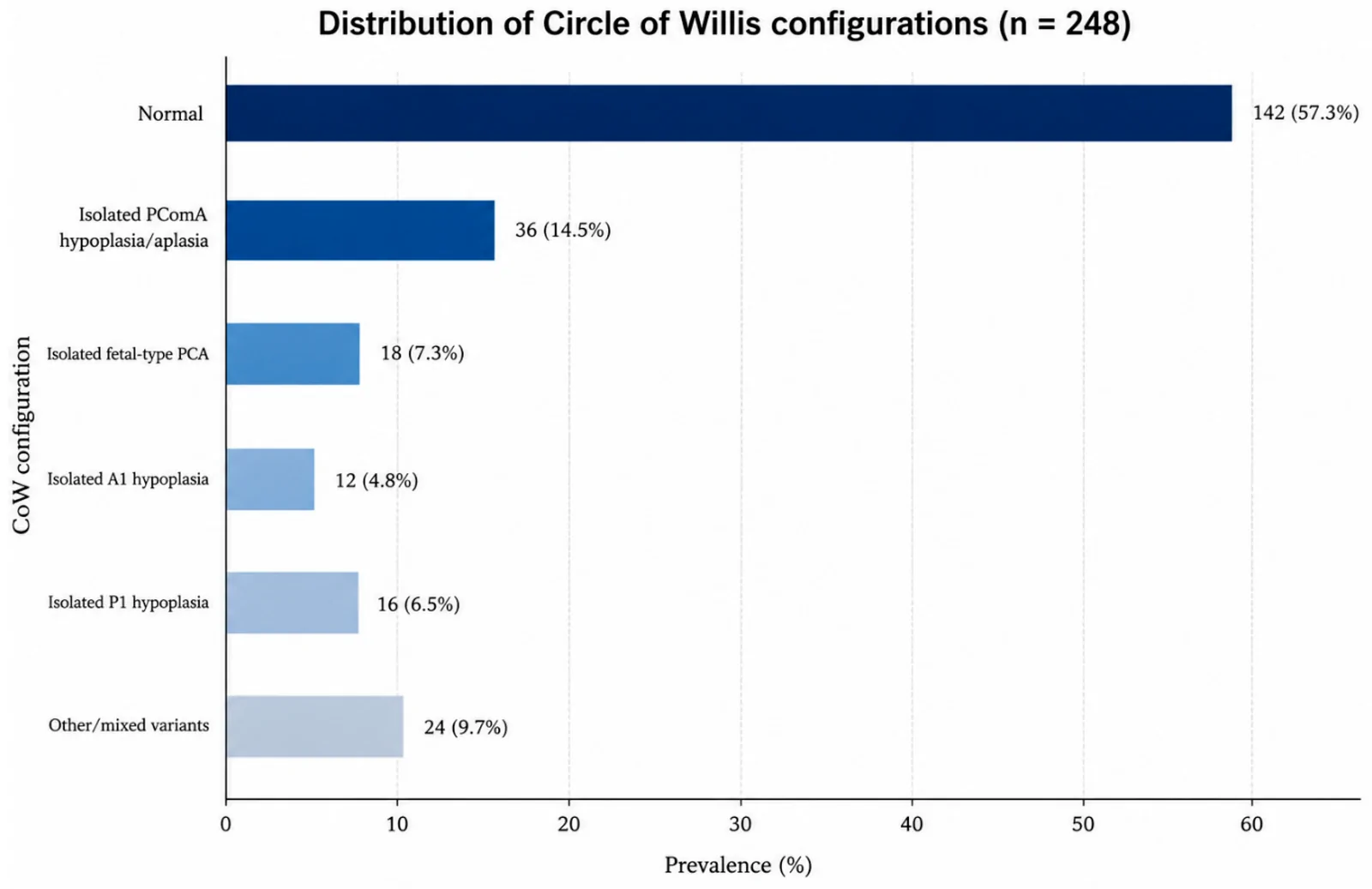
Distribution of mutually exclusive patient-level CoW configurations in the study cohort (*n* = 248). Bars represent the number and percentage of patients assigned to each configuration category.

A normal CoW configuration was identified in 142 patients (57.3%), whereas 106 patients (42.7%) had at least one anatomical variant. In the mutually exclusive patient-level classification (Table 2, Panel A), isolated PComA hypoplasia/aplasia was the most frequent variant (*n* = 36, 14.5%), followed by isolated fetal-type PCA (*n* = 18, 7.3%), isolated P1 hypoplasia (*n* = 16, 6.5%), and isolated A1 hypoplasia (*n* = 12, 4.8%). Other isolated rare or mixed configurations were identified in 24 patients (9.7%). The distribution of the mutually exclusive CoW configurations is illustrated in Figure 2.

In the non-exclusive component analysis (Table 2, Panel B), PComA hypoplasia/aplasia involvement was the most frequent component (*n* = 47, 19.0%), followed by fetal-type PCA (*n* = 20, 8.1%), P1 segment hypoplasia (*n* = 17, 6.9%), A1 segment hypoplasia (*n* = 14, 5.6%), and AComA hypoplasia/aplasia involvement (*n* = 11, 4.4%). Less frequent components included X-shaped ACA (*n* = 5, 2.0%), ACA trifurcation (*n* = 3, 1.2%), vascular duplication (*n* = 2, 0.8%), azygos ACA (*n* = 1, 0.4%), and other PCA anomaly (*n* = 1, 0.4%). Because the component-level analysis was non-exclusive, patients with mixed configurations could contribute to more than one variant category.

### 3.2. Comparison of Normal and Variant Configurations

Patients with a variant CoW configuration were significantly older than those with a normal configuration [median, 52.0 years (IQR, 38.2–58.0) vs. 39.0 years (IQR, 36.0–50.0); *p* < 0.001]. Migraine was more frequent in the variant group than in the normal group (24.5% vs. 4.9%; *p* < 0.001), as was diabetes mellitus (16.0% vs. 5.6%; *p* = 0.007). Conversely, a history of stroke/TIA (27.4% vs. 56.3%; *p* < 0.001) and carotid stenosis (11.3% vs. 26.1%; *p* = 0.004) were less frequent among patients with variant CoW configurations. No statistically significant between-group differences were observed for sex, hypertension, smoking, or hyperlipidemia (Table 3).

### 3.3. Configuration-Specific Exploratory Comparisons

Nine global exploratory tests compared the six mutually exclusive CoW configuration groups, including all 24 patients with other/mixed variants (Table 4). Age, stroke/TIA history, migraine, carotid stenosis, and hypertension each had a reported *p*-value <0.001 and met the Bonferroni threshold of 0.05/9 (approximately 0.0056). Diabetes mellitus had a reported *p*-value of 0.006 and did not meet this multiplicity-controlled threshold, although it was below the nominal 0.05 level. Sex (*p* = 0.305), smoking (*p* = 0.159), and hyperlipidemia (*p* = 0.580) did not meet either threshold. These global tests do not establish differences between particular pairs of configurations. Migraine was recorded in 13 of 16 patients (81.2%) with isolated P1 hypoplasia, compared with 7 of 142 (4.9%) with normal anatomy; these descriptive proportions are based on markedly unequal group sizes. No post hoc pairwise testing was performed.

### 3.4. Logistic Regression and Model Performance

**Figure 3:**
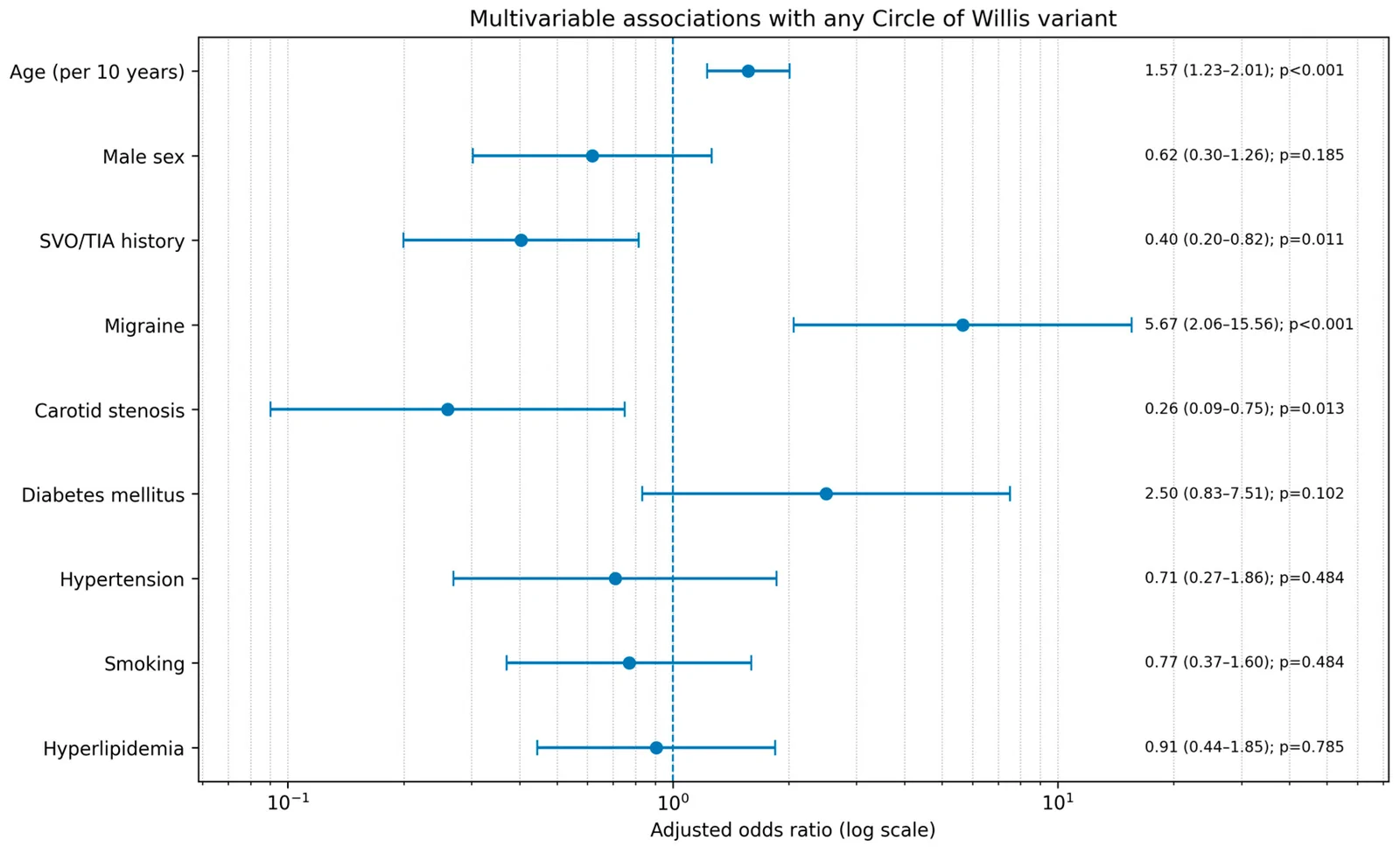
Forest plot of adjusted odds ratios (ORs) and 95% confidence intervals (CIs) for factors associated with the presence of any CoW variant in the multivariable logistic regression model. The dashed vertical line indicates the null value (OR = 1.00).

In univariate analyses, older age, migraine, and diabetes mellitus were associated with higher odds of having any CoW variant, whereas a history of stroke/TIA and carotid stenosis were inversely associated with the presence of a variant (Table 5). In the multivariable model, each 10-year increase in age was associated with higher odds of a CoW variant (aOR, 1.57; 95% CI, 1.23–2.01; *p* < 0.001), as was migraine (aOR, 5.67; 95% CI, 2.06–15.56; *p* < 0.001). In contrast, a history of stroke/TIA (aOR, 0.40; 95% CI, 0.20–0.82; *p* = 0.011) and carotid stenosis (aOR, 0.26; 95% CI, 0.09–0.75; *p* = 0.013) remained inversely associated with the presence of a CoW variant. Diabetes mellitus was no longer statistically significant after multivariable adjustment (aOR, 2.50; 95% CI, 0.83–7.51; *p* = 0.102) (Figure 3).

**Figure 4:**
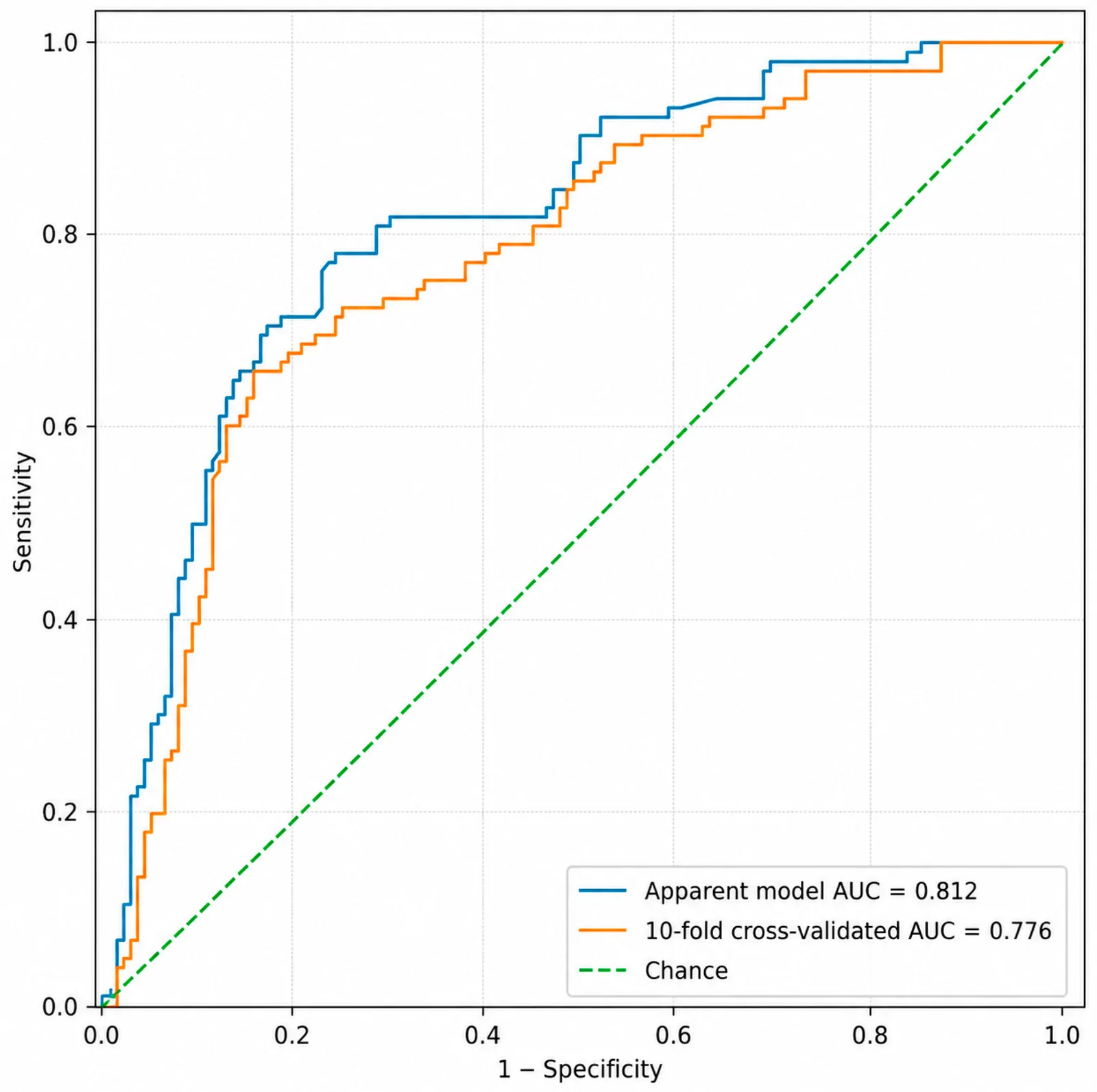
Receiver operating characteristic (ROC) curves for the multivariable logistic regression model for the presence of any CoW variant. The apparent model AUC was 0.812, and the 10-fold cross-validated AUC was 0.776. The diagonal dashed line represents chance-level discrimination (AUC = 0.50).

The multivariable model was statistically significant overall (likelihood-ratio χ^2^ = 68.45, df = 9; *p* < 0.001), with a Nagelkerke R^2^ of 0.324. The apparent AUC was 0.812 (bootstrap 95% CI, 0.756–0.865), while the 10-fold cross-validated AUC was 0.776 (Figure 4). The Brier score was 0.177. The Hosmer–Lemeshow goodness-of-fit test was significant (χ^2^ = 16.10, df = 8; *p* = 0.041), suggesting suboptimal calibration. Accordingly, despite its acceptable discriminatory performance, the model was interpreted as an explanatory association model rather than as a validated clinical prediction tool.

### 3.5. Representative CTA Findings

**Figure 5:**
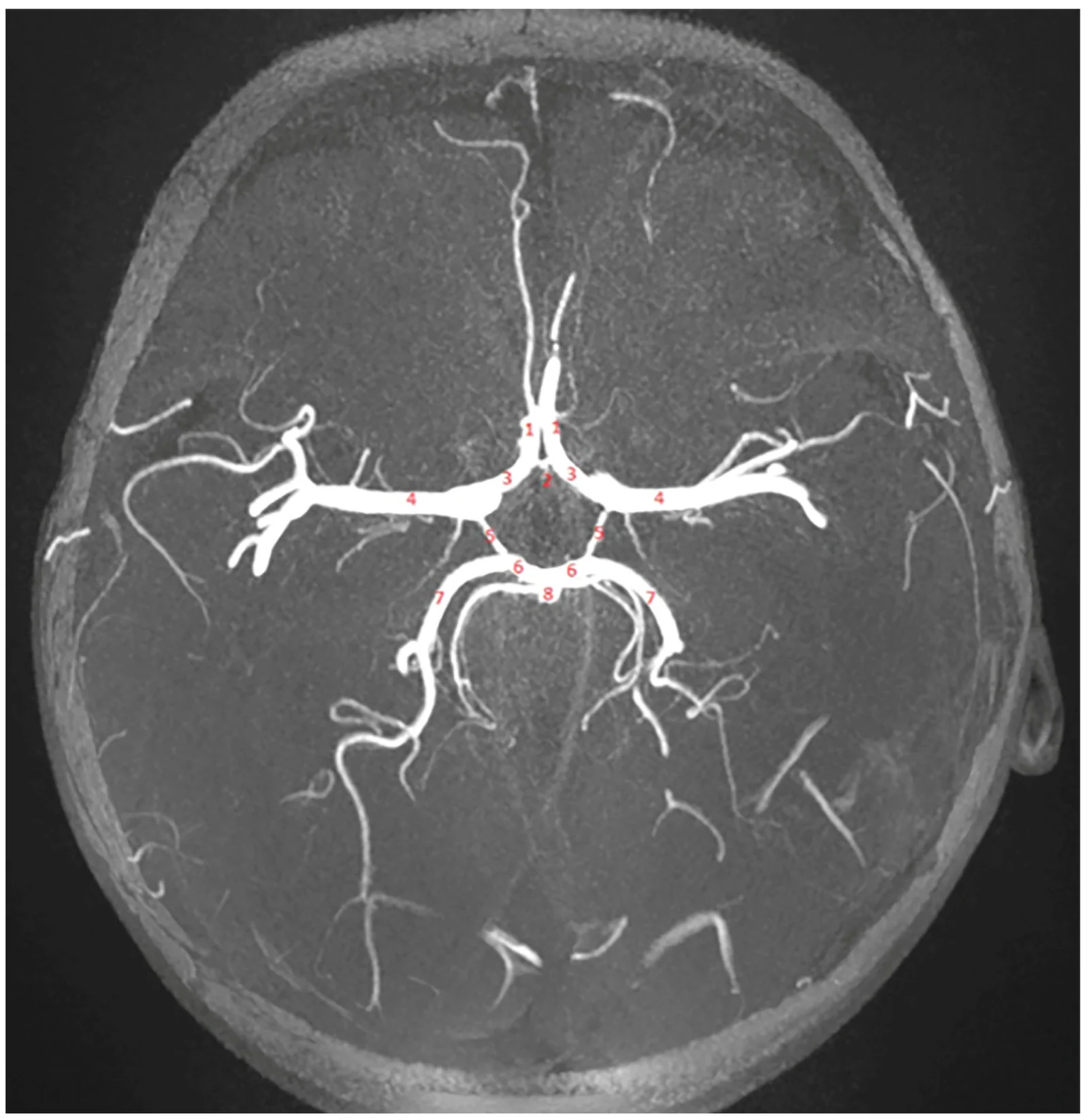
Representative angiographic image of a normal CoW configuration. 1, A2 segment of the ACA; 2, AComA; 3, A1 segment of the ACA; 4, MCA; 5, PComA; 6, P1 segment of the PCA; 7, P2 segment of the PCA; 8, basilar artery.

**Figure 6:**
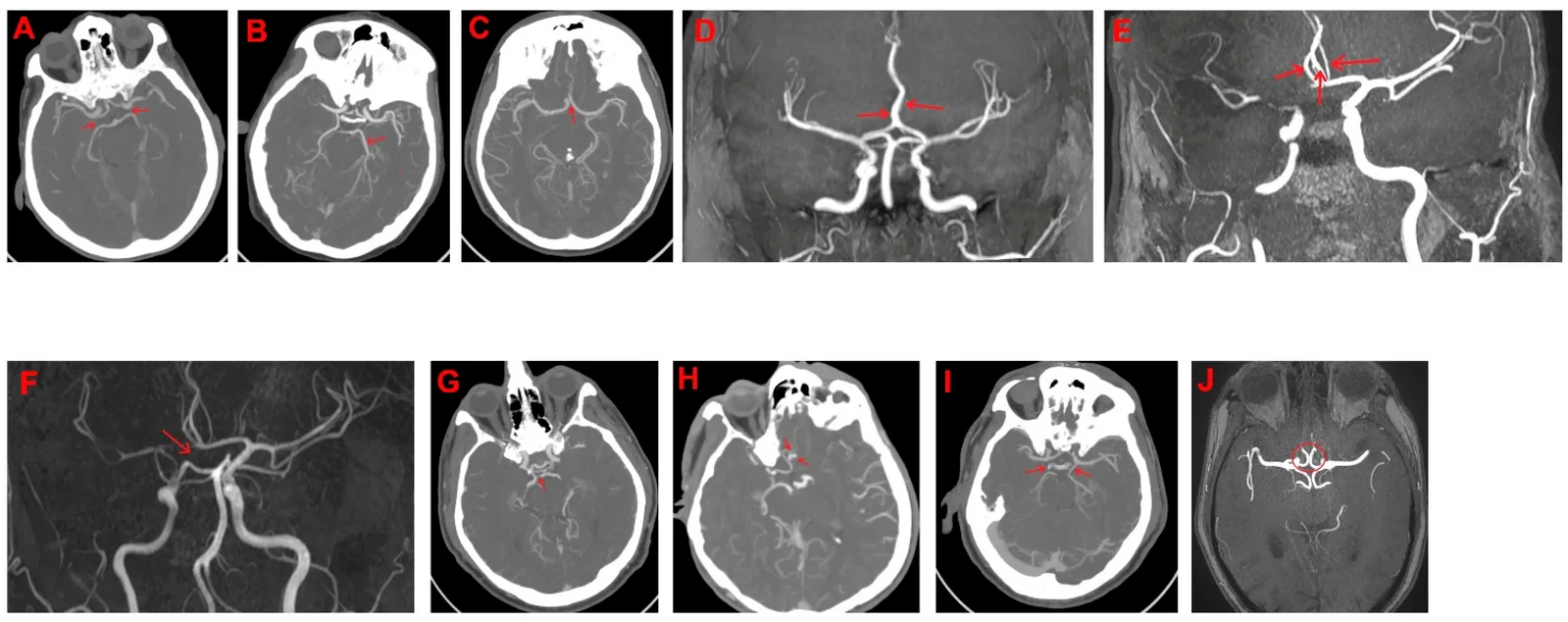
Representative CTA images illustrating anatomical variants of the CoW. (**A**) Bilateral CTA-defined aplasia of the PComAs; arrows indicate the expected locations of the non-visualized communicating segments. (**B**) Fetal-type PCA configuration with a prominent PComA and a relatively small ipsilateral P1 segment; the arrow identifies the fetal-type configuration. (**C**) CTA-defined aplasia of the AComA; the arrow indicates the expected location of the non-visualized connection between the anterior cerebral circulations. (**D**) Azygos ACA configuration; arrows identify the single midline A2 trunk. (**E**) ACA trifurcation; arrows indicate the three distal ACA branches arising from the AComA complex. (**F**) CTA-defined aplasia of the A1 segment; the arrow indicates the expected location of the non-visualized segment. (**G**) P1 segment hypoplasia; the arrow identifies the reduced-caliber segment. (**H**) AComA duplication; arrows indicate the two distinct communicating channels. (**I**) Mixed posterior circulation configuration comprising a fetal-type left PCA and CTA-defined aplasia of the right PComA; arrows indicate the corresponding findings. (**J**) X-shaped ACA configuration; the circled region highlights the crossing arrangement of the anterior cerebral arterial segments. CTA-defined aplasia denotes non-visualization on CTA and does not independently confirm congenital absence. ACA, anterior cerebral artery; AComA, anterior communicating artery; CoW, Circle of Willis; CTA, computed tomography angiography; PCA, posterior cerebral artery; PComA, posterior communicating artery.

Representative CTA appearances of the normal CoW configuration and major anatomical variants are presented in Figure 5 and Figure 6, respectively. Figure 5 shows a normal CoW configuration, with preservation of the principal arterial components and communicating segments. The labeled structures include the bilateral A2 segments of the anterior cerebral arteries (ACAs), AComA, bilateral A1 segments, middle cerebral arteries (MCAs), PComAs, P1 and P2 segments of the PCAs, and the basilar artery.

The representative CTA examples in Figure 6 illustrate the anatomical diversity underlying the patient-level and component-level classifications. The findings involve both anterior and posterior circulation components and include variations in vessel caliber, CTA-defined non-visualization, arterial branching, and communicating connections. Anterior circulation examples demonstrate differences in the number and arrangement of arterial channels (AComA aplasia, azygos ACA, ACA trifurcation, A1 aplasia, and AComA duplication; Figure 6C–F,H), whereas posterior circulation examples illustrate asymmetric communicating pathways and fetal-type PCA anatomy (PComA aplasia, fetal-type PCA, and P1 hypoplasia; Figure 6A,B,G). An X-shaped ACA arrangement is also shown (Figure 6J).

The composite figure includes isolated findings and a mixed posterior circulation configuration (fetal-type left PCA with CTA-defined right PComA aplasia; Figure 6I), demonstrating that multiple arterial variants can coexist in the same patient. Such configurations are represented by a single category in the mutually exclusive patient-level analysis, while each involved component is recorded separately in the non-exclusive component-level analysis. CTA-defined aplasia refers to non-visualization of the relevant segment and does not independently establish congenital absence.

Table 6 contextualizes the classification framework and prevalence findings of the present study. Panel A summarizes major anatomical classification systems, from cadaveric- and autopsy-based descriptions to imaging-based approaches and the comprehensive framework of Ayre et al., which catalogued 82 variants [5]. Panel B presents selected CTA-based prevalence studies alongside the present cohort, distinguishing anterior-component, posterior-component, and whole-circle completeness from normal configurations. The comparison highlights differences in classification endpoints and assessment methods that must be considered when interpreting prevalence estimates across studies.

## 4. Discussion

This study characterized CoW anatomy using cranial CTA in 248 patients and extended the descriptive prevalence analysis by evaluating demographic and clinical correlates of variant configurations. A variant CoW configuration was identified in 42.7% of the cohort, with PComA hypoplasia/aplasia representing both the most frequent isolated variant and the most frequently involved arterial component when mixed configurations were also considered. Older age and migraine remained associated with higher odds of any CoW variant after multivariable adjustment, whereas a history of stroke/TIA and carotid stenosis showed inverse associations. The multivariable model demonstrated acceptable discrimination but limited calibration, supporting its interpretation as an explanatory association model rather than a clinical prediction tool.

The 57.3% prevalence of a normal CoW configuration in this cohort is higher than the complete-configuration estimates reported in the Turkish CTA and Norwegian population studies discussed above [12,19]. These contrasts should be interpreted in relation to the present clinical referral sample, its younger average age than the Tromsø cohort, and differences in imaging and classification criteria. Even studies from the same country have produced markedly different estimates [12,16]. Moreover, a normal configuration in our classification is not necessarily equivalent to every published definition of a complete circle. Our findings therefore characterize patients undergoing CTA at one Turkish center and do not establish an anatomical pattern attributable to Turkish ethnicity. Age- and sex-comparable cohorts assessed with harmonized imaging and definitions are needed for meaningful geographic comparisons.

Posterior circulation variants predominated in the present cohort. Isolated PComA hypoplasia/aplasia occurred in 14.5% of patients, while PComA involvement was identified in 19.0% when mixed configurations were included. Although higher frequencies of PComA hypoplasia or aplasia have been reported in some studies, direct comparisons are complicated by differences in imaging resolution, definitions of hypoplasia, and whether bilateral or mixed configurations are counted separately [1,2,3,4,16,17,19,22]. Because the PComAs constitute important collateral pathways between the anterior and posterior circulations, accurate recognition of their presence and morphology remains relevant to cerebrovascular assessment and interventional planning [6,9,12,22].

Fetal-type PCA was identified in 7.3% of patients as an isolated configuration and in 8.1% in the non-exclusive component analysis, within the broad range reported in previous studies [17,23]. Isolated P1 and A1 hypoplasia occurred in 6.5% and 4.8% of patients, respectively, whereas their corresponding component frequencies were 6.9% and 5.6%. AComA hypoplasia/aplasia involvement was identified in 4.4%. X-shaped ACA configuration, ACA trifurcation, vascular duplication, azygos ACA, and other PCA anomalies were less frequent. These findings also illustrate the importance of distinguishing mutually exclusive patient-level configurations from non-exclusive component-level prevalence, particularly because patients with mixed configurations may contribute to more than one anatomical component category.

Population representativeness and individual anatomical heterogeneity are distinct methodological considerations. Representative recruitment addresses whether a cohort reflects the demographic and geographic distribution of the population of interest. Component-level coding addresses differences in arterial configuration among individuals, including those within the same demographic group. This distinction parallels methodological arguments in Alzheimer’s neuroimaging and neurological rehabilitation that separate population diversity from individual biological heterogeneity [24,25]. Here, mutually exclusive patient-level categories summarize overall configurations, while non-exclusive component coding retains coexisting arterial findings. Detailed coding cannot correct selection bias, and broader recruitment alone cannot recover anatomical information lost through a binary outcome. Both considerations should therefore inform future CoW studies.

Migraine showed the strongest positive clinical association with variant CoW anatomy in the adjusted analysis (aOR, 5.67; 95% CI, 2.06–15.56). This finding is directionally consistent with prospective MRA evidence linking migraine with aura to incomplete CoW configurations [7], whereas a case–control study of migraine without aura found no corresponding association [26]. Relationships with fetal-type posterior circulation patterns have also been investigated [23,27]. However, only 33 participants had migraine: 26 had variant anatomy, and 7 had a normal configuration. Migraine and carotid stenosis were recorded in relatively small proportions of the cohort (13.3% and 19.8%, respectively), warranting cautious interpretation of both associations. The wide confidence interval indicates limited precision, and sparse, unevenly distributed observations may produce an unstable or exaggerated point estimate. The 81.2% migraine frequency in the isolated P1 group represents only 13 of 16 patients and does not establish a P1-specific association. The migraine analysis did not include aura status, attack frequency, chronicity, or treatment information. The magnitude of the adjusted association therefore requires replication in larger, clinically characterized migraine subgroups.

Each 10-year increase in age was associated with 57% higher adjusted odds of a CTA-defined variant configuration. The Tromsø Study also reported more apparently missing arterial segments with increasing age, while recognizing that TOF MRA visibility can depend on flow [19]. In our CTA cohort, differences in vessel caliber, acquired narrowing, contrast opacification, and referral patterns are possible contributors; these explanations were not tested. Developmental variants should not be interpreted as arising de novo with advancing age. The cohort included pediatric and adult patients aged 0–94 years; therefore, the pooled coefficient cannot be interpreted specifically as an aging effect within adults without age-stratified evaluation. The model assumes a common linear age association with the log odds; it does not distinguish pediatric–adult differences, nonlinear age effects, or longitudinal change. Adult-only analyses and flexible age models would help clarify this finding. Sex, hypertension, smoking, and hyperlipidemia were not significantly associated with variant anatomy after adjustment.

A history of stroke/TIA and carotid stenosis were inversely associated with variant CoW anatomy in the multivariable analysis. Importantly, these findings should not be interpreted as evidence that variant or incomplete CoW anatomy is protective. Previous literature has frequently associated incomplete CoW configurations with reduced collateral capacity and cerebrovascular ischemic risk [8,9,10,28]. The inverse associations observed in the present cohort may instead reflect selection and indication biases inherent to a retrospective CTA-based clinical sample, the exclusion of patients with acute ischemic stroke at the time of imaging, differences in the clinical characteristics of patients undergoing CTA, or residual confounding. Given the cross-sectional nature of the analysis, temporal relationships and causality cannot be established.

The apparent AUC of 0.812 and 10-fold cross-validated AUC of 0.776 indicate that the model discriminated between normal and variant configurations, with some optimism in the apparent performance. The significant Hosmer–Lemeshow test (*p* = 0.041) raises a separate concern about agreement between estimated probabilities and observed frequencies. One possible explanation is the aggregation of anatomically heterogeneous variants into a single outcome: migraine prevalence, for example, ranged from 8.3% in isolated PComA hypoplasia/aplasia to 81.2% in isolated P1 hypoplasia. If clinical associations differ by configuration, a common set of regression coefficients may inadequately describe the combined outcome. Sparse subgroups, unmeasured clinical factors, and the assumed linear age term could also contribute. Neither the Hosmer–Lemeshow result nor these descriptive differences establish the cause of miscalibration. Calibration plots, intercept and slope estimates, and external validation would be needed to characterize it. The model should be used to explore associations and should not guide individual clinical decisions.

High-resolution CTA facilitates the recognition of arterial configurations relevant to image interpretation and procedural planning [12,13,22]. Future studies should retain component-level coding and assess whether component-specific or multinomial models better describe clinical associations when sample sizes permit. These analyses would require adequate numbers within each configuration, prespecified comparisons, and validation of calibration as well as discrimination. Combining anatomical classification with quantitative perfusion measurements and longitudinal outcomes would help determine whether specific variants modify collateral function or clinical risk.

**Figure 7:**
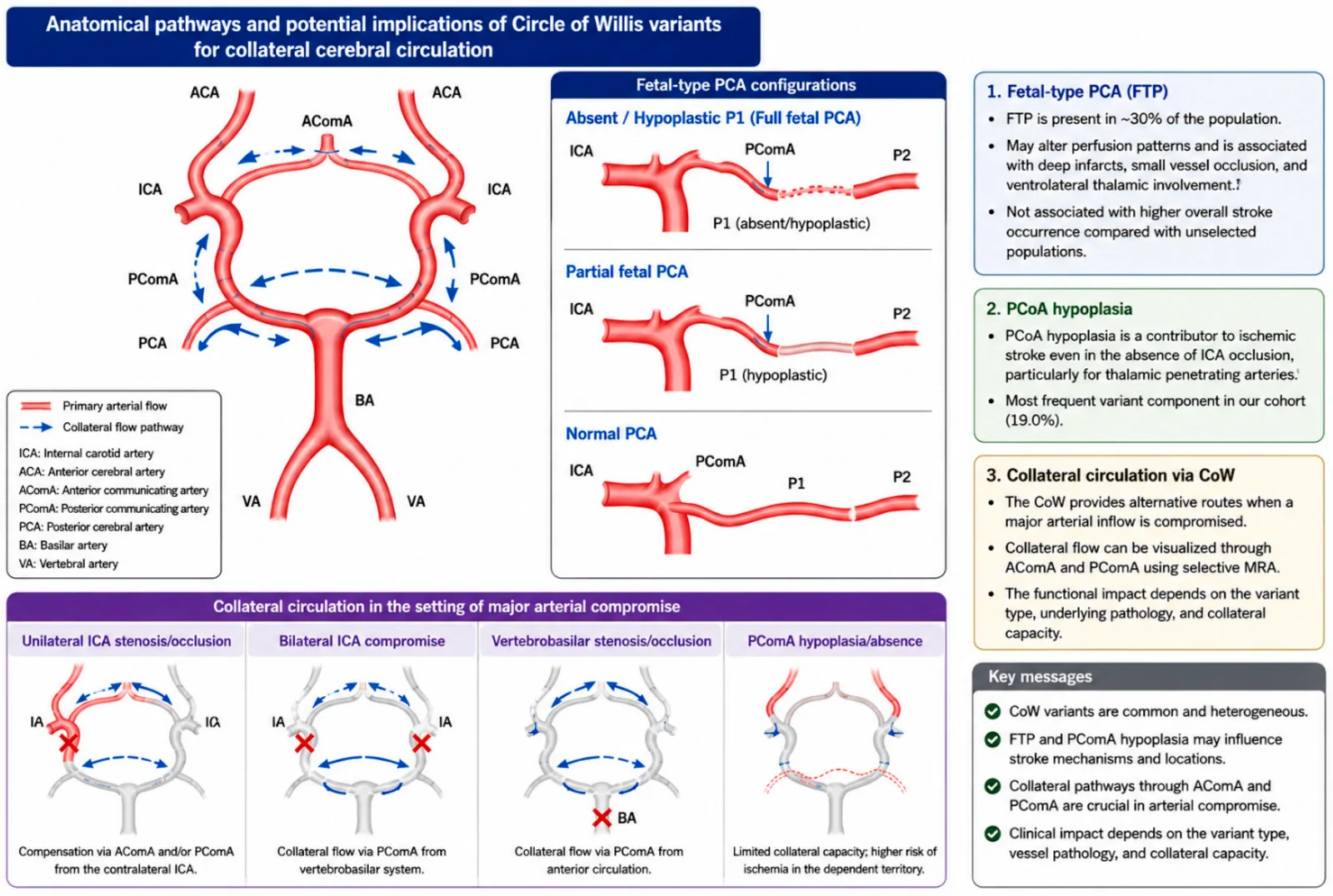
Schematic representation of the anatomical pathways and potential implications of major CoW variants for cerebral collateral circulation. Fetal-type posterior cerebral artery and posterior communicating artery hypoplasia may modify collateral pathways, while the functional consequences of these configurations depend on the affected arterial segment, underlying vascular pathology, and available collateral capacity.

The potential clinical relevance of CoW variants extends beyond their morphological classification because the communicating arteries provide alternative routes for cerebral blood flow when primary arterial inflow is compromised (Figure 7). This concept is particularly relevant to the posterior circulation variants that predominated in our cohort. Davidoiu et al. evaluated 139 CT angiograms and demonstrated considerable morphological heterogeneity among fetal-type PCA configurations, including unilateral and bilateral forms and their coexistence with additional anterior and posterior CoW variants [29]. This observation complements our findings, in which fetal-type PCA was identified in 7.3% of patients as an isolated configuration and in 8.1% in the non-exclusive component analysis, emphasizing the importance of considering fetal-type PCA within the overall CoW configuration rather than solely as an isolated vascular feature [29].

The relationship between fetal-type PCA and cerebrovascular disease appears to be complex rather than uniformly adverse. Frid et al. evaluated 1407 patients with MRI-DWI-confirmed acute ischemic stroke and 546 patients from an unselected hospital population and found a similar prevalence of fetal-type PCA configurations in the two groups (31% and 32%, respectively). Furthermore, the presence of an ipsilateral fetal-type PCA was not associated with PCA-territory infarction [30]. In contrast, Ryu and Kim evaluated 394 patients with PCA-territory stroke and reported differences in infarct patterns and stroke mechanisms according to fetal-type PCA status; patients with fetal-type PCA more frequently exhibited deep infarction, small-vessel occlusion, and ventrolateral thalamic involvement [31]. Taken together, these observations suggest that fetal-type PCA may influence cerebral perfusion architecture and patterns of ischemic injury under specific clinical conditions without necessarily being associated with a higher overall occurrence of ischemic stroke [30,31]. This distinction is relevant to our cohort, in which fetal-type PCA was relatively common, but the cross-sectional design did not permit evaluation of variant-specific stroke mechanisms.

The predominance of PComA abnormalities may also have functional relevance. Chuang et al. reported PComA hypoplasia in 19.35% of 310 patients with acute ischemic stroke and 8.20% of matched controls, with an association even in the absence of ICA occlusion (OR, 3.21; 95% CI, 1.43–9.62) [32]. PComA involvement occurred in 47 patients (19.0%) in our component-level analysis. Among the 36 patients with isolated PComA hypoplasia/aplasia, 15 (41.7%) had a stroke/TIA history, compared with 80 of 142 patients (56.3%) with normal anatomy. These descriptive counts do not establish a component-specific association because the full PComA-positive subgroup also includes 11 patients with mixed configurations. Our binary regression combined different variants, excluded acute ischemic stroke at CTA, and did not assess previous stroke by type, vascular territory, or mechanism. Thus, it cannot resolve whether associations differ between ischemic and hemorrhagic stroke, TIA, or etiological subtypes, and it should not be directly equated with an acute ischemic stroke study.

The functional importance of CoW anatomy may become particularly evident when a major arterial inflow pathway is compromised. Ito et al. demonstrated in patients with cervical ICA stenosis that selective MRA could visualize collateral blood flow through both the anterior and posterior communicating arteries, with findings corresponding closely to those obtained using DSA [33]. These observations provide functional support for the role of the CoW as a collateral network rather than solely an anatomical structure [33]. Accordingly, the inverse association between carotid stenosis and CoW variants observed in our cohort should not be interpreted as evidence of a protective effect of variant anatomy. Instead, the available evidence supports a more nuanced framework in which the clinical consequences of a particular CoW configuration may depend on the arterial component involved, the underlying vascular pathology, and the capacity for collateral recruitment. The principal anatomical pathways and their potential implications for cerebral collateral circulation are summarized schematically in Figure 7.

For clinical reporting, the relevant information includes which communicating pathways are available, whether abnormalities are unilateral or bilateral, and whether posterior cerebral territories receive a dominant ICA contribution through a fetal-type PCA [6,29,33]. These features may affect interpretation of ischemic territories and the anatomical assessment undertaken before neurovascular procedures. A visible arterial connection alone does not establish sufficient collateral flow. Our study did not measure perfusion, collateral recruitment, procedural outcomes, or subsequent cerebrovascular events, so the observed variants cannot be assigned individual treatment or prognostic consequences.

Vascular contributions to neurological syndromes also warrant consideration. Dunalska et al. reviewed corticobasal syndrome associated with cerebrovascular pathology, illustrating that vascular injury can produce a clinical syndrome commonly investigated in a neurodegenerative context [34]. This raises the hypothesis that limited collateral reserve in certain CoW configurations could modify vulnerability to chronic hypoperfusion when other vascular disease is present. Neither that review nor our study demonstrates that CoW variants cause neurodegeneration. Testing this hypothesis would require prospective assessment of arterial anatomy, perfusion, vascular disease burden, and longitudinal cognitive or neurodegenerative outcomes.

Several limitations constrain interpretation. First, this retrospective single-center CTA referral cohort is not a population-based sample. Its male predominance (58.9%), broad pediatric–adult age range (0–94 years), and clinical case mix limit extrapolation to the general population. Ethnicity, ancestry, and regional demographic composition were not characterized in the reported analysis, so recruitment in Türkiye should not be equated with ethnic or national representativeness. The inclusion of pediatric and adult patients, together with the absence of age-stratified estimates, prevents separation of pediatric–adult differences from aging effects within adults. Exclusion of acute ischemic stroke may also distort associations with previous stroke/TIA and carotid stenosis. Detailed anatomical coding preserves individual configurations but does not remedy these sampling limitations.

Second, CTA non-visualization cannot reliably distinguish congenital aplasia from very small vessels, inadequate opacification, or acquired narrowing. The <1-mm threshold and imaging resolution may lead to misclassification and limit comparisons with MRA, DSA, or cadaveric studies. Although disagreements were resolved by consensus, an independent interobserver agreement statistic was not reported. Anatomical continuity was assessed without measurements of collateral flow or cerebrovascular reserve. The present study therefore provides an anatomical CTA-based assessment rather than a functional evaluation of collateral circulation. Anatomical completeness does not necessarily correspond to functional collateral capacity. Future studies incorporating TCD, dynamic arterial spin labeling MRI, or other perfusion-based imaging approaches could provide complementary information on flow patterns, tissue perfusion, and the functional consequences of specific configurations.

Third, the migraine subgroup and several configuration groups were small, yielding imprecise estimates and possible sparse-data bias. The carotid stenosis subgroup was also limited in size, comprising 49 patients, only 12 of whom had variant anatomy. Accordingly, both the positive migraine association and the inverse carotid stenosis association should be regarded as exploratory, cohort-specific findings requiring independent replication. Although a Bonferroni threshold was applied to the nine global configuration comparisons, these analyses remain exploratory and do not establish differences between specific pairs of configurations. The diabetes comparison did not meet this multiplicity-controlled threshold. Other reported clinical comparisons and regression associations were interpreted at nominal significance levels and require independent confirmation. Clinical variables were largely binary; the analyses did not characterize migraine aura, stroke type or mechanism, event timing, disease severity, or pharmacotherapy. Medication-related and other residual confounding therefore remain possible. The combined stroke/TIA variable cannot establish subtype-specific associations.

Finally, the binary outcome combines configurations that may have different functional consequences. The regression model showed suboptimal calibration, used a common linear age term, and lacked external validation. Cross-validation of discrimination does not resolve these limitations. No longitudinal perfusion, cognitive, or clinical outcome data were analyzed; consequently, temporal relationships, causal mechanisms, and prognostic or neurodegenerative implications cannot be established.

## 5. Conclusions

At least one CoW variant was identified in 42.7% of this Turkish CTA cohort. PComA hypoplasia/aplasia was the most frequent isolated configuration (14.5%) and the most common arterial component when mixed configurations were included (19.0%). Combined patient-level and component-level reporting captures coexisting anatomical findings and provides information relevant to mapping potential collateral pathways, interpreting vascular imaging, and planning neurovascular procedures. Future multicenter studies should combine representative recruitment with standardized component-level coding, evaluate age-defined groups, characterize migraine subtypes, pharmacotherapy, and stroke mechanisms, and link arterial anatomy to functional perfusion and longitudinal outcomes. Independent validation of component-specific associations and model performance would further clarify the clinical significance of these configurations.

## Figures and Tables

**Table 1:** Demographic and clinical characteristics of the study population.

Variable	Overall (*n* = 248)
Age, years, median (IQR)	47.0 (36.0–54.0)
Age, years, mean ± SD	45.7 ± 15.9
Age range, years	0–94
Male sex	146 (58.9)
Female sex	102 (41.1)
Stroke/TIA history	109 (44.0)
Migraine	33 (13.3)
Carotid stenosis	49 (19.8)
Diabetes mellitus	25 (10.1)
Hypertension	74 (29.8)
Smoking	55 (22.2)
Hyperlipidemia	68 (27.4)

Values are presented as *n* (%) unless otherwise specified. Age is presented as median (IQR) and mean ± SD. IQR, interquartile range; SD, standard deviation; TIA, transient ischemic attack.

**Table 2:** Distribution of CoW configurations and anatomical variant components.

Classification	*n* (%)
Panel A. Mutually exclusive patient-level classification	
Normal configuration	142 (57.3)
Isolated PComA hypoplasia/aplasia	36 (14.5)
Isolated fetal-type PCA	18 (7.3)
Isolated A1 hypoplasia	12 (4.8)
Isolated P1 hypoplasia	16 (6.5)
Other isolated rare or mixed variants	24 (9.7)
Total	248 (100.0)
Panel B. Non-exclusive component-level classification	
PComA hypoplasia/aplasia	47 (19.0)
Fetal-type PCA	20 (8.1)
AComA hypoplasia/aplasia	11 (4.4)
Azygos ACA	1 (0.4)
ACA trifurcation	3 (1.2)
A1 hypoplasia	14 (5.6)
P1 hypoplasia	17 (6.9)
Vascular duplication	2 (0.8)
X-shaped ACA	5 (2.0)
Other PCA anomaly	1 (0.4)

Values are presented as *n* (%). Panel A represents a mutually exclusive patient-level classification, with each patient assigned to a single CoW configuration. Panel B represents a non-exclusive component-level classification; therefore, patients with mixed configurations may contribute to more than one variant component. Percentages in both panels were calculated using the total study population (*n* = 248) as the denominator. ACA, anterior cerebral artery; AComA, anterior communicating artery; CoW, Circle of Willis; PCA, posterior cerebral artery; PComA, posterior communicating artery.

**Table 3:** Comparison of demographic and clinical characteristics between patients with normal and variant CoW configurations.

Variable	Normal CoW (*n* = 142)	Variant CoW (*n* = 106)	*p*-Value
Age, years, median (IQR)	39.0 (36.0–50.0)	52.0 (38.2–58.0)	<0.001
Male sex	88 (62.0)	58 (54.7)	0.251
Stroke/TIA history	80 (56.3)	29 (27.4)	<0.001
Migraine	7 (4.9)	26 (24.5)	<0.001
Carotid stenosis	37 (26.1)	12 (11.3)	0.004
Diabetes mellitus	8 (5.6)	17 (16.0)	0.007
Hypertension	46 (32.4)	28 (26.4)	0.309
Smoking	32 (22.5)	23 (21.7)	0.875
Hyperlipidemia	36 (25.4)	32 (30.2)	0.398

Values are presented as *n* (%) unless otherwise specified. Age is presented as median (IQR). The Mann–Whitney U test was used for comparison of age, and Pearson’s chi-square test was used for categorical variables. IQR, interquartile range; CoW, Circle of Willis; TIA, transient ischemic attack.

**Table 4:** Comparison of demographic and clinical characteristics across mutually exclusive CoW configuration groups.

Variable	Normal (*n* = 142)	PComA (*n* = 36)	Fetal PCA (*n* = 18)	A1 (*n* = 12)	P1 (*n* = 16)	Other/Mixed (*n* = 24)	*p*-Value
Age, years, median (IQR)	39.0 (36.0–50.0)	55.0 (38.5–76.0)	44.5 (24.0–53.8)	53.5 (47.8–56.2)	49.5 (41.0–53.2)	54.0 (36.2–58.0)	<0.001
Male sex	88 (62.0)	18 (50.0)	13 (72.2)	7 (58.3)	6 (37.5)	14 (58.3)	0.305
Stroke/TIA history	80 (56.3)	15 (41.7)	4 (22.2)	1 (8.3)	0 (0.0)	9 (37.5)	<0.001
Migraine	7 (4.9)	3 (8.3)	5 (27.8)	2 (16.7)	13 (81.2)	3 (12.5)	<0.001
Carotid stenosis	37 (26.1)	3 (8.3)	0 (0.0)	0 (0.0)	7 (43.8)	2 (8.3)	<0.001
Diabetes mellitus	8 (5.6)	9 (25.0)	1 (5.6)	0 (0.0)	3 (18.8)	4 (16.7)	0.006
Hypertension	46 (32.4)	16 (44.4)	2 (11.1)	0 (0.0)	0 (0.0)	10 (41.7)	<0.001
Smoking	32 (22.5)	4 (11.1)	4 (22.2)	5 (41.7)	6 (37.5)	4 (16.7)	0.159
Hyperlipidemia	36 (25.4)	10 (27.8)	4 (22.2)	6 (50.0)	5 (31.2)	7 (29.2)	0.580

Values are presented as *n* (%) unless otherwise specified. Age is presented as median (IQR). Age was compared across groups using the Kruskal–Wallis test, whereas categorical variables were compared using the Fisher–Freeman–Halton exact test with 100,000 Monte Carlo samples. The six configuration groups are mutually exclusive and include the other/mixed group (*n* = 24), accounting for all 248 patients. PComA, fetal PCA, A1, and P1 columns represent isolated configurations. The displayed *p*-values are the reported global test values, not Bonferroni-adjusted *p*-values. Multiplicity was addressed by comparison with the threshold 0.05/9 (approximately 0.0056); no pairwise comparisons were performed. A1, A1 segment hypoplasia; CoW, Circle of Willis; IQR, interquartile range; P1, P1 segment hypoplasia; PCA, posterior cerebral artery; PComA, posterior communicating artery; TIA, transient ischemic attack.

**Table 5:** Univariate logistic regression analysis of factors associated with the presence of any CoW variant.

Variable	Unadjusted OR	95% CI	*p*-Value
Age (per 10-year increase)	1.35	1.13–1.60	<0.001
Male sex	0.74	0.44–1.24	0.251
Stroke/TIA history	0.29	0.17–0.50	<0.001
Migraine	6.27	2.60–15.10	<0.001
Carotid stenosis	0.36	0.18–0.74	0.005
Diabetes mellitus	3.20	1.32–7.73	0.010
Hypertension	0.75	0.43–1.31	0.309
Smoking	0.95	0.52–1.75	0.875
Hyperlipidemia	1.27	0.73–2.23	0.399

The dependent variable was the presence of any CoW variant (variant = 1; normal configuration = 0). ORs were estimated using univariate binary logistic regression. Age was modeled per 10-year increase. CI, confidence interval; CoW, Circle of Willis; OR, odds ratio; TIA, transient ischemic attack.

**Table 6:** Anatomical classification frameworks of the CoW and selected CTA-based prevalence studies.

**Panel A.** Anatomical classification frameworks				
**Classification and Year**	**Authors and Study Design**	**Method and Sample**	**Configurations and Main Features**	**Anatomical and Clinical Relevance**
Lazorthes (1979) [4]	G. Lazorthes et al.; anatomical study	Cadaver dissection; 305 cadavers	22 subtypes; symmetry and patency of AComA, A1, PComA, and P1; includes A1 hypoplasia, unilateral PComA absence, and AComA duplication	Framework for describing communicating pathways and anatomy relevant to aneurysm surgery
Riggs and Rupp (1963) [3]	H.E. Riggs and C. Rupp; autopsy study	Autopsy dissection; 994 adult brains	21 types; segmental hypoplasia, aplasia, and discontinuity; includes fetal-type PCA and absent communicating segments	Anatomical basis for considering potential collateral pathways
Krabbe-Hartkamp (1998) [2]	M.J. Krabbe-Hartkamp et al.; prospective imaging study	3D TOF MRA; 150 individuals	Classification of anterior and posterior configurations according to completeness and symmetry	Non-invasive characterization of CoW anatomy and component completeness
Alpers (1959) [20]	B.J. Alpers, R.G. Berry, and R.M. Paddison; autopsy study	Autopsy examination; 350 normal brains	Descriptions of fetal-type PCA, AComA morphology, and A1 variations	Foundational reference for anatomical variation
Yaşargil (1984) [21]	M.G. Yaşargil; microsurgical atlas	Microsurgical anatomical assessment; cohort size not applicable	Surgically relevant configurations, including fetal-type PCA, hypoplastic A1, and absent PComA	Anatomical guidance for neurovascular surgery
Ayre et al. (2022) [5]	J.R. Ayre et al.; systematic review	Literature-based anatomical synthesis	82 variants; hypoplasia, aplasia, accessory arteries, duplication, and anomalous connections	Comprehensive framework for standardized anatomical description and coding
**Panel B.** Selected CTA-based prevalence studies				
**Study**	**Population and Sample**	**Imaging Method**	**Classification or Reported Endpoint**	**Principal Findings**
Karataş et al. (2015) [12]	Turkish cohort; 100 patients	Cranial CTA	Complete polygonal configuration without hypoplasia	Complete configuration: 28/100 (28%)
Li et al. (2011) [14]	Chinese cohort; 160 patients in the final analysis	64-section multidetector CTA	Separate assessment of anterior, posterior, and whole-circle completeness	Complete anterior component: 126/160 (79%); complete posterior component: 50/160 (31%); complete whole circle: 43/160 (27%); fetal-type posterior configuration: 15/160 (9.4%)
Present study	Turkish clinical referral cohort; 248 pediatric and adult patients	Cranial CTA	Mutually exclusive patient-level configurations and non-exclusive component-level coding; hypoplasia defined as luminal diameter < 1 mm; CTA-defined aplasia as non-visualization	Normal configuration: 142/248 (57.3%); any variant: 106/248 (42.7%); isolated PComA hypoplasia/aplasia: 36/248 (14.5%); overall PComA involvement: 47/248 (19.0%)

Panel A summarizes anatomical classification frameworks; panel B provides a structured comparison of selected CTA studies rather than an exhaustive systematic review. Anterior-component completeness, posterior-component completeness, whole-circle completeness, and normal configuration are distinct endpoints. Differences in recruitment, age distribution, imaging technique, vessel-diameter thresholds, and classification criteria limit direct comparison of prevalence estimates. Anatomical completeness does not establish functional collateral capacity. CTA-defined aplasia in the present study denotes non-visualization rather than independently confirmed congenital absence. Abbreviations: A1, precommunicating segment of the anterior cerebral artery; ACA, anterior cerebral artery; AComA, anterior communicating artery; CoW, Circle of Willis; CTA, computed tomography angiography; MRA, magnetic resonance angiography; P1, precommunicating segment of the posterior cerebral artery; PCA, posterior cerebral artery; PComA, posterior communicating artery; TOF, time-of-flight.

## Data Availability

The datasets generated and/or analyzed during the current study are available from the corresponding author upon reasonable request.

## Abbreviations

ACA	anterior cerebral artery
AComA	anterior communicating artery
aOR	adjusted odds ratio
AUC	area under the receiver operating characteristic curve
CI	confidence interval
CoW	Circle of Willis
CTA	computed tomography angiography
DSA	digital subtraction angiography
ICA	internal carotid artery
IQR	interquartile range
MCA	middle cerebral artery
MIP	maximum intensity projection
MPR	multiplanar reconstruction
MRA	magnetic resonance angiography
OR	odds ratio
PCA	posterior cerebral artery
PComA	posterior communicating artery
ROC	receiver operating characteristic
SD	standard deviation
TIA	transient ischemic attack
TOF	time-of-flight
VIF	variance inflation factor
